# Metabolites managing excess manganese: The *SgPAL2*-regulated flavonoids in stylo

**DOI:** 10.1093/plphys/kiaf062

**Published:** 2025-02-25

**Authors:** Munkhtsetseg Tsednee

**Affiliations:** Assistant Features Editor, Plant Physiology, American Society of Plant Biologists; Agricultural Biotechnology Research Center, Academia Sinica, Taipei 11529, Taiwan

Plants require manganese (Mn) to support many biological processes such as respiration and photosynthesis. Mn is a cofactor of various enzymes, for example, Mn-superoxide dismutase (Alejandro et al. 2020). However, in excess, Mn causes oxidative stress, impaired photosynthesis, and disrupted enzyme activity, thereby reducing plant growth and productivity (Alejandro et al. 2020).

In acidic soil, Mn toxicity occurs commonly due to abundant presence of bioavailable Mn form (Mn^2+^) (Shao et al. 2017). Considering that 40% to 50% of the world's arable lands are acidic, located mainly in tropical and subtropical areas (Kochian et al. 2004), Mn toxicity challenges plant growth and development, especially crop production. But some plants, such as tropical legume stylo (*Stylosanthes guianensis*), tolerate excess Mn and adapt to acidic soils (Chen et al. 2015). Uncovering how these plants cope with Mn toxicity could provide insights into improving plant tolerance to excess Mn.

In *Plant Physiology*, Wang et al. (2025) investigated the Mn tolerance mechanisms in stylo genotypes and identified a metabolic regulatory gene and metabolites involved in Mn detoxification. Initially, the authors confirmed and worked with a Mn-tolerant genotype, RY5, that showed no defects in root growth and enhanced antioxidant activity in high Mn. These observations suggested that RY5 uses the internal Mn detoxification mechanism to tolerate high Mn contents.

Previously, specialized metabolites have been suggested to be involved in Mn tolerance in different plant species, for example, cowpea (Führs et al. 2012) and citrus (Zheng et al. 2024). Indeed, over 230 Mn-responsive metabolites could be identified in RY5 exposed to elevated Mn. Of these, the majority were flavonoids and phenolic compounds.

The authors used their earlier transcriptomic analyses in stylo with Mn supply (Jia et al. 2020) and looked at the mRNA accumulations of genes related to the phenylpropanoid/flavonoid pathway in both RY5 and TF0317 roots. From the RY5-specific genes, the authors then selected *PHENYLALANINE AMMONIA-LYASE 2* (*SgPAL2*), whose expression increased over 4-fold in response to excess Mn, for its functional study.

Phenylalanine ammonia-lyase is a key enzyme in the phenylpropanoid pathway and catalyzes the conversion of phenylalanine to trans-cinnamic acid (Zhang and Liu 2015). PAL activity increased in RY5 roots, but not in TF0317 roots, in excess Mn treatment, and the inhibition of PAL activity decreased Mn tolerance in RY5. These results indicated the potential role of *SpPAL2* in conferring Mn tolerance in the RY5 genotype.

To confirm the *SpPAL2* function, Wang et al. (2025) further generated *OX-SgPAL2* overexpression and *RNAi-SpPAL2* knockdown lines. Although all of the lines showed similar levels of Mn accumulation, the *OX-SgPAL2* lines tolerated the high Mn (Fig. 1A) compared with the RNAi knockdown lines. Moreover, the antioxidant enzyme activities and 16 genes related to the biosynthesis of secondary metabolites significantly increased in *OX-SgPAL2* overexpression lines. Together, these results demonstrated that *SgPAL2* functions in Mn tolerance in stylo, with an anticipated role in metabolic regulations.

**Figure 1.**
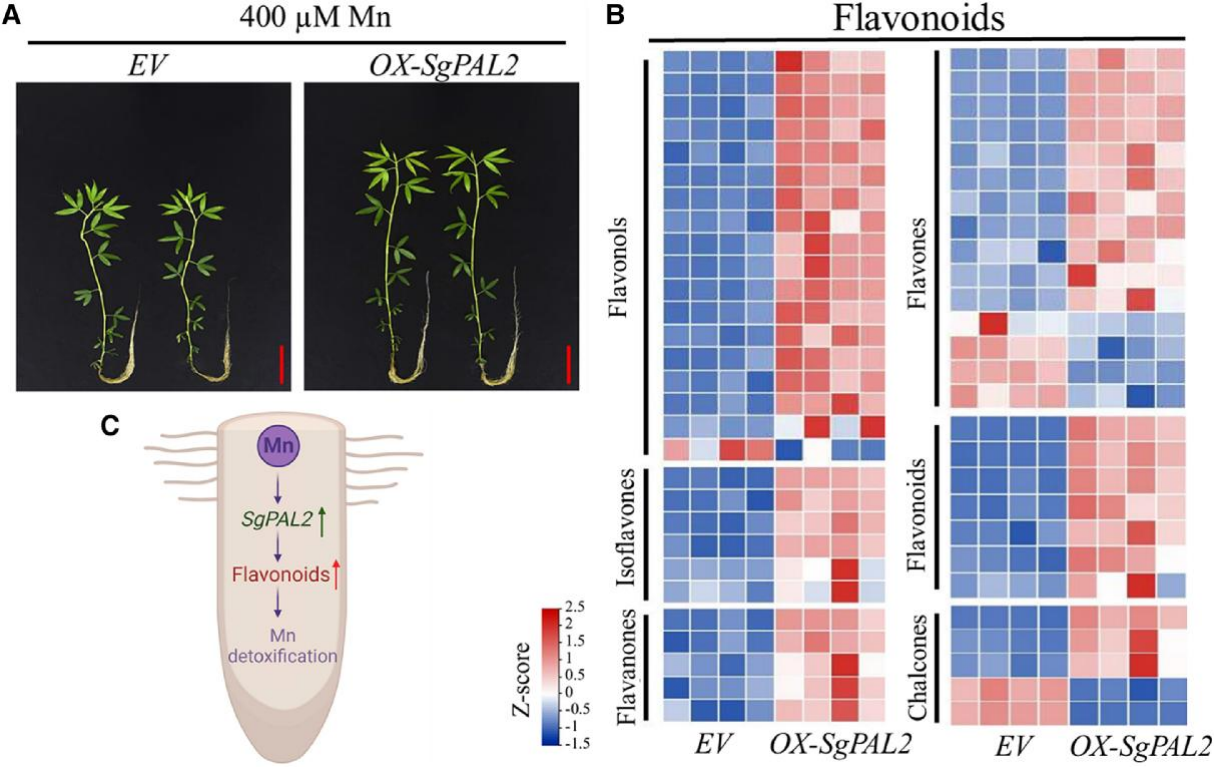
Growth of *OX-SpPAL2* overexpression stylo lines with enriched flavonoid accumulations and a conceptual diagram of *SpPAL2* regulation for Mn detoxification. **A)** Effects of *SpPAL2* overexpression on stylo growth under excess Mn treatment. The transgenic stylo plants, with empty vector control (*EV*) and *SpPAL2* overexpression (*OX-SgPAL2*), were grown in Hoagland culture solution containing 400 *µ*M MnSO4 (pH 5.0) for 14 days. Bar = 4 cm. **B)** Heatmap analysis of differentially accumulated metabolites (DAMs) associated with flavonoids in transgenic hairy roots regulated by *SgPAL2* overexpression. Four biological replicates of transgenic plants were included in the experiment (modified from Wang et al. (2025)). **C)** The simplified conceptual diagram representing the *SpPAL2-*regulated flavonoid functions in Mn detoxification in stylo roots.

Lastly, to identify the *SpPAL2-*regulated metabolites, the authors performed metabolomic analyses in *OX-SpPAL2* overexpression versus control lines and observed the enrichments of metabolites from the phenylpropanoid/flavonoid pathway in the overexpression lines (Fig. 1B). They further obtained 25 candidate metabolites, including 4 flavonoids, those abundances were also increased in RY5 roots under high Mn.

An exogenous supply of one of these candidate flavonoids, calycosin, enhanced the stylo root growth under excess Mn and decreased the ROS level triggered by excess Mn. Thus, the results confirmed that the *SpPAL2-*regulated calycosin flavonoid achieves Mn detoxification by reducing ROS accumulation in stylo roots (Fig. 1C).

In summary, Wang et al. (2025) have identified the critical metabolic regulatory gene, *SpPAL2*, and metabolites associated with Mn tolerance in stylo. This finding advances the understanding of excess metal detoxification and tolerance mechanisms in plants. Moreover, the study provides a promising target and route to improve plant tolerance to excess Mn through engineering the phenylpropanoid/flavonoid pathway.

## Data Availability

No data associated with this article.
